# Systemic drug-related intertriginous and flexural exanthema induced by the Pfizer-BioNTech COVID-19 vaccine: A report of 2 cases

**DOI:** 10.1016/j.jdcr.2021.10.016

**Published:** 2021-10-26

**Authors:** Josephine Hai, Harrison Shawa, Penelope Kim-Lim, Jenny Z. Wang, Michelle Vy, Maxwell A. Fung, Apra Sood, Danielle M. Tartar

**Affiliations:** a Department of Dermatology, University of California, Davis, Sacramento, California; b Kirk Kerkorian School of Medicine at the University of Nevada, Las Vegas, Nevada; c Department of Pathology and Laboratory Medicine, University of California, Davis, Sacramento, California; d Sacramento VA Medical Center, Sacramento, California

**Keywords:** coronavirus, SDRIFE, vaccine, SDRIFE, systemic drug-related intertriginous and flexural exanthema

## Introduction

Systemic drug-related intertriginous and flexural exanthema (SDRIFE) is a well-demarcated erythematous dermatitis observed after systemic drug exposure. The dermatitis is typically symmetrically distributed with predominance in the intertriginous and/or flexural areas.^1^ The prognosis is typically good after cessation of the causative agent, and topical or systemic steroids can be used as needed to accelerate symptom resolution.

This report discusses 2 patients in whom SDRIFE-like reactions developed after they received the Pfizer-BioNTech COVID-19 vaccine (BNT162b2; Comirnaty). We considered medications, vaccine components, and COVID-19 infection as potential causative agents. Both patients experienced significant improvement without significant sequelae with corticosteroid treatment.

## Case reports

### Case 1

A severely pruritic and painful eruption developed in a healthy 23-year-old man 6 weeks after his second dose of the Pfizer-BioNTech COVID-19 vaccine. The rash began on his hips and progressed to his neck, axillae, gluteal folds, thighs, and buttocks, eventually becoming confluent on his back as well. Other than vaccination, he had no other changes to his medications, which consisted only of fish oil and vitamin E supplements. He denied other changes in topical products or other recent exposure.

One month after rash onset, he presented to the emergency department with uncontrolled pain and itching of the rash. He had seen various other providers and had been prescribed topical ketoconazole, triamcinolone, and oral terbinafine, with no improvement.

**Fig 1:**
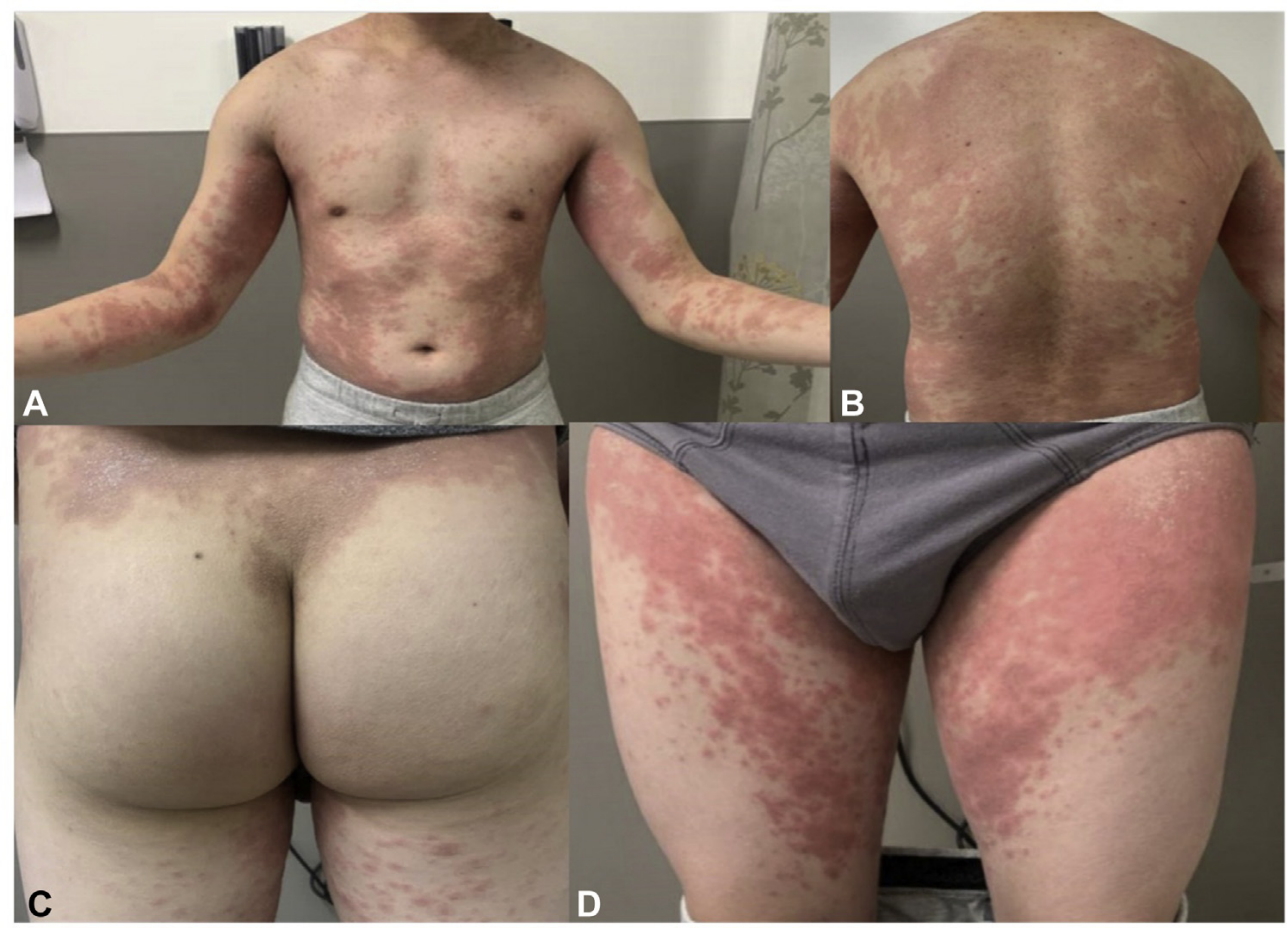
Dusky-red scaly papules coalescing into confluent plaques on the (**A**) chest, trunk, and arms; (**B**) back; (**C**) buttocks; and (**D**) inguinal area and thighs of patient 1.

**Fig 2:**
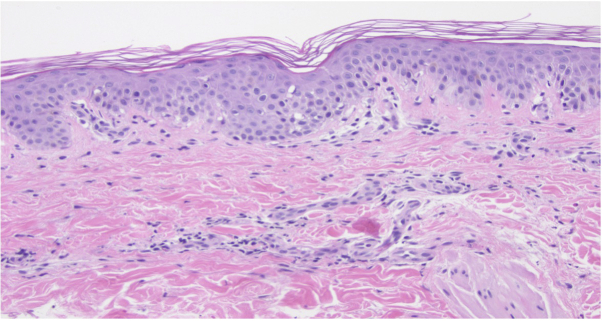
Biopsy taken laterally on the right thigh of patient 1 revealing vacuolar interface dermatitis with mild spongiosis and eosinophils. (Hematoxylin-eosin stain; original magnification: ×10.)

The patient denied having systemic symptoms. He was otherwise well-appearing, and his vital signs were within normal limits. Skin examination was significant for dusky-red scaly papules coalescing into confluent plaques favoring the intertriginous and flexural surfaces of his extremities, back, and chest (Fig 1, *A*-*D*). The complete metabolic panel was within normal limits, but the complete blood cell count was significant for elevated eosinophils (1,700 cells/μL). Punch biopsies from 2 sites (laterally on the right thigh and right upper back) showed vacuolar interface dermatitis with mild spongiosis (Fig 2) and negative direct immunofluorescence. The combined presence of vacuolar interface changes and numerous eosinophils along with eosinophilic spongiosis favored a drug reaction, namely SDRIFE, given his clinical presentation. The patient was diagnosed with SDRIFE in response to the COVID-19 vaccine. He was educated on the natural course of the disease and prescribed topical clobetasol. Over the next month, his rash completely resolved.

### Case 2

A 38-year-old woman presented with a rash that started 2 weeks after her second dose of the Pfizer-BioNTech COVID-19 vaccine. The rash started on the nape of the neck and progressed to the entire neck, behind the ears, axillae, flexural forearms, and groin, later spreading to the scalp and upper chest. She denied any systemic symptoms. Her past medical history was significant for depression, and she had been receiving paroxetine 30 mg daily for many years. The medication was temporarily discontinued by her primary care provider, with no effect on the progression of the rash. She denied taking any over-the-counter supplements or any changes in personal care products. Comprehensive patch testing to the American Contact Dermatitis Society Core 80 showed a mild 1+ reaction to nickel. The patient had a history of intolerance to cheap jewelry and denied any recent exposure to nickel or any changes in her diet.

**Fig 3:**
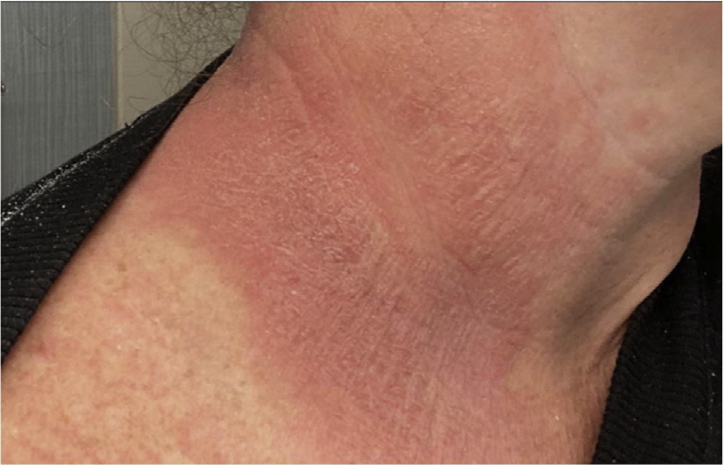
Erythematous, scaly plaques on the neck of patient 2.

The patient was otherwise well, with stable vital signs. On examination, she had involvement of the neck, retroauricular areas, upper back, upper chest, axillae, and antecubital fossae with well-demarcated erythematous scaly plaques (Fig 3). Additionally, there was mild erythema and edema of her upper eyelids. A skin biopsy from the neck revealed spongiotic dermatitis with eosinophils and negative direct immunofluorescence. The complete blood cell count and complete metabolic panel were within normal limits, and antinuclear antigen and extranuclear antigen were negative. Her COVID-19 test was negative.

She was given a 9-day prednisone taper starting at 40 mg and desonide cream twice daily for the face and neck with gradual improvement in the rash. Based on the clinical and histopathologic presentation, a diagnosis of SDRIFE to the Pfizer-BioNTech COVID-19 vaccine was made.

## Discussion

The pathogenic mechanism of SDRIFE is not well understood, but it is thought to be most likely due to a type IV delayed hypersensitivity immune response.^2^ Although SDRIFE is uncommon, cases in the literature document SDRIFE caused by antibiotics, asthma medications, radiocontrast media, chemotherapeutic agents, and biologics.^3^

To date, there has been only one other report of SDRIFE associated with a COVID-19 vaccine.^4^ In this report, SDRIFE developed 4 days after the patient received the CoronaVac vaccine (it is unknown whether it was the first or the second dose) developed by SinoVac Biotech. Vaccine components shared between CoronaVac and the Pfizer-BioNTech vaccine include sodium chloride and disodium hydrogen phosphate, both of which are inactive ingredients.^4^^,^^5^ (Table I). To our knowledge, there are no previous reports of any SDRIFE-like eruptions associated with these vaccine components, which are shared by many standard vaccines, including hepatitis B and influenza vaccines. The only other cases of SDRIFE associated with vaccines involve the mercury-based preservative thimerosal,^6^ an inactive ingredient that is not present in either the CoronaVac or the Pfizer-BioNTech vaccine. In considering possible contact dermatitis, nickel is also not a component in either vaccine (Table I). Thus, the pathophysiology of COVID-19 vaccine-associated SDRIFE remains to be elucidated. The timeline of the reactions described in this report suggests a delayed hypersensitivity response, similar to that seen in drug reaction with eosinophilia and systemic symptoms, although our patients did not have systemic symptoms. In fact, drug reaction with eosinophilia and systemic symptoms resulting from COVID-19 vaccination has previously been reported.^7^

**Table I:** Ingredients of the CoronaVac vaccine manufactured by SinoVac Biotech and of the Pfizer-BioNTech vaccine, also known as BNT162b2. (Active ingredients are in bold text at the top of the columns, inactive ingredients in plain text make up the rest of the columns. Shared ingredients between both COVID-19 vaccines are highlighted in gray.)

CoronaVac^4^	Pfizer-BioNTech vaccine (BNT162b2)^5^
**Inactivated SARS-CoV-2 virus**	**Spike glycoprotein mRNA of SARS-CoV-2**
Aluminum hydroxide	Monobasic potassium phosphate
Disodium hydrogen phosphate	Disodium hydrogen phosphate
Monosodium hydrogen phosphate	Potassium chloride
Sodium chloride	Sodium chloride
Sodium hydroxide	Sucrose
	(4-hydroxybutyl)azanediyl)bis(hexane-6,1-diyl)bis(2-hexyldecanoate)
	2 [(polyethylene glycol)-2000]-N,N-ditetradecylacetamide
	1,2-Distearoyl-sn-glycero-3- phosphocholine
	Cholesterol

Given the growing reports of COVID-19 cutaneous manifestations, we also considered infection as the trigger of SDRIFE in both of our patients. For SDRIFE specifically, there are already a few reported cases associated with symptomatic COVID-19 infection.^3^^,^^8^^,^^9^ However, in some of these reports, it is unclear whether the SDRIFE-like eruption could be due to COVID-19 itself or the drugs used to treat the COVID-19 infection. In our presented cases, the absence of documented COVID-19 infection and systemic symptoms favors a reaction to the vaccine rather than to infection.

Aside from the unusual length of time between exposure and rash onset, the 2 patients in this report had the typical clinical and histopathologic features of SDRIFE following COVID-19 vaccination. Although SDRIFE usually has a latency period of a few hours to days, in some cases, onset after a few weeks has been reported.^1^^,^^10^ Because the period of latency can be quite variable, it is not one of the criteria typically used in making the diagnosis of SDRIFE. The authors would like to emphasize that SDRIFE typically has a good prognosis after appropriate treatment. Both patients improved significantly with corticosteroid treatment.

## Conflicts of interest

None disclosed.
